# What is public health advocacy? Australasian practitioner perspectives on defining the field and identifying effective practice and required competencies

**DOI:** 10.1093/heapro/daag150

**Published:** 2026-10-21

**Authors:** Alexandra J Bhatti, Sophia Lin, Katherine Cullerton, Dannielle Post, Nathan Dawes, Janaki Amin, Josephine Y Chau

**Affiliations:** School of Health Sciences and Nursing, Macquarie University, 75 Talavera Rd, Sydney, NSW 2019, Australia; School of Health Sciences, UNSW Sydney, Level 6 North, Health Transition Hub, 55 Botany St Randwick, Sydney, NSW 2031, Australia; School of Public Health, The University of Queensland, Public Health Building, 288 Herston Rd, Herston, QLD 4006, Australia; School of Public Health, Adelaide University, Level 4, Rundle Mall Plaza, 50 Rundle Mall, Adelaide, SA 5000, Australia; College of Medicine and Dentistry, James Cook University, 1 James Cook Drive, Townsville, QLD 4811, Australia; School of Population Health, UNSW Sydney, Level 5, Health Translation Hub, 55 Botany St. Randwick, Sydney, NSW 2031, Australia; Health Protection NSW, NSW Health, 1 Reserve Rd, St Leonards, NSW 2065, Australia; School of Health Sciences and Nursing, Macquarie University, 75 Talavera Rd, Sydney, NSW 2019, Australia

**Keywords:** advocacy, public health advocacy, advocacy practice, advocacy evaluation, public health workforce, advocacy competencies

## Abstract

Public health advocacy is a core function of public health practice, yet it remains underexamined, with limited consensus on its definition, effective strategies, and required competencies. This study explored public health advocacy practice among Australasian advocates, examining how advocacy is defined, which strategies are perceived as effective, and the competencies required for practice. Public health advocates completed an online survey between December 2024 and April 2025. Data were analysed using descriptive statistics and content analysis. A total of 101 advocates completed the survey. Public health advocacy was commonly defined as an intentional, outcomes-focused practice that uses strategic actions to influence policy and practice to improve population health. Relationship building (59%), influencing decision makers (57%), and media use (46%) were identified as the most effective strategies. Reported barriers included inadequate organizational resourcing (funding 63%, time 61%, personnel 47%), limited access to decision makers (56%), organizational concerns about repercussions (39%), low priority (32%), and insufficient training (32%). Required competencies included contextual understanding of the health issue, policymaking literacy, knowledge of advocacy methods, and skills in communication, collaboration, research, and media engagement. Australasian advocates hold broadly consistent views of public health advocacy and identify key competencies and effective strategies. However, barriers related to capacity, organizational support, and evaluation processes continue to constrain practice. Strengthening workforce capability requires targeted education and training, greater organizational prioritization and resourcing of advocacy, and the development of strong relational networks with stakeholders and decision makers to enhance advocacy effectiveness. These findings have relevance beyond Australasia, particularly in comparable high-income democratic countries.

Contribution to Health Promotion

- Despite the important role of advocacy in health promotion, there is limited empirical evidence describing how it is conceptualized and practiced by the public health workforce.
- This study provides practitioner-informed evidence on how Australasian advocates define public health advocacy, the strategies identified as effective, the competencies required for practice, and the barriers that constrain advocacy.
- The findings identify competencies that can inform health promotion education, competency frameworks, and professional development to strengthen advocacy capacity.
- Strengthening advocacy capacity through education and organizational investment can enhance policy influence and support more effective health promotion action in Australasia and other comparable income settings.

## Background

Advocacy is a core function of health promotion and public health practice, as it plays a vital role in translating evidence into action to improve population health (World Health Organization 1986, Flavel *et al*. 2024). Its significance is reflected in global frameworks that guide public health practice, including the World Health Organizations (WHO) Ottawa Charter for Health Promotion (World Health Organization 1986.) and the Global Charter for Public Health (World Federation of Public Health Associations 2024). Developed by the WHO and World Federation of Public Health Associations, the Global Charter identifies advocacy as one of four enablers that support the delivery of the three core public health services of protection, prevention, and promotion (World Federation of Public Health Associations 2024). Despite this recognized importance, public health advocacy is a relatively underexamined area within the broader public health field (Cullerton *et al*. 2026).

Additionally, there is a lack of consensus among practitioners, academics, and advocacy organizations regarding the meaning and scope of public health advocacy (Cohen and Marshall 2017, Smith and Stewart 2017, Schneider *et al*. 2025). Existing definitions reflect diverse conceptualizations of advocacy and vary in their emphasis on advocacy goals, processes, and intended outcomes, reflecting the broad range of activities and purposes associated with advocacy. Definitions differ in whether advocacy focuses on influencing policy and structural change, community empowerment, or individual behaviour change, and whether it involves strategically influencing public and political agendas or working collaboratively with communities to identify and address their priorities, contributing to the ongoing uncertainty about the boundaries of public health advocacy. Consistent with these findings, an Australian study of public health educators identified substantial diversity and ambiguity in how advocacy is understood, including variation in its scope and interpretation (Bhatti *et al*. 2026). This ambiguity can limit clarity regarding the competencies (the integration of knowledge, skills, and attributes) required for advocacy practice. Advocacy competencies are recognized as essential for public health professionals in Australasia (Council of Academic Public Health Institutions Australasia 2016) and internationally (World Health Organization 2020, The Council on Linkages between Academia and Public Health Practice 2021, National Collaborating Centres for Public Health 2025) and form part of accreditation requirements for health promotion practitioners (Battel-Kirk *et al*. 2024). However, existing frameworks vary in the extent to which they articulate these competencies, with advocacy capabilities often implied rather than explicitly specified (Hearne and Jernigan 2024). For example, advocacy is identified as a distinct competency domain within both the WHO European public health competency framework and International Union for Health Promotion and Education (IUHPE) competencies, whereas the Australasian public health competency framework embeds advocacy within competency elements and underpinning knowledge, providing less explicit guidance on advocacy competencies. Consequently, there is no universally agreed set of competencies to guide advocacy practice (Blundell *et al*. 2023), education (Bhatti *et al*. 2022, Schneider *et al*. 2024), or workforce development (O'Connell *et al*. 2016). With the Australasian public health competency framework undergoing review at the time of this study, there was a timely opportunity to strengthen how advocacy competencies are articulated. Consistent with this, public health professionals feel underprepared to engage effectively in advocacy practice (Schneider *et al*. 2025, Cullerton *et al*. 2026). Greater clarity regarding the competencies required for advocacy is therefore needed to inform education and training initiatives, support professional development, and strengthen workforce capacity (Sykes *et al*. 2023, Hearne and Jernigan 2024).

Despite these challenges, public health professionals report regularly undertaking advocacy activities (Kerr *et al*. 2017, Cullerton *et al*. 2026), yet assessment of the effectiveness of these activities remains limited (Garcia *et al*. 2015, Blenner *et al*. 2017, Cohen and Marshall 2017, Smith and Stewart 2017, Košir 2024), constraining the development of evidence-informed practice and reducing the potential impact of advocacy efforts.

Research indicates advocates face additional contextual barriers that can limit the effectiveness of their practice, including gaps in advocacy skills and preparedness (Sainkhuu *et al*. 2021, Sykes *et al*. 2023, Cullerton *et al*. 2026), limited time and funding (Cullerton *et al*. 2026), low organizational prioritization of advocacy (Cullerton *et al*. 2026), and opposition from powerful industries whose commercial interests often conflict with public health goals (Cullerton *et al*. 2016). Academic advocates may also work within performance systems that have not traditionally recognized advocacy-related activities to the same extent as publications and grant funding. Much of the existing evidence originates from international contexts, with a paucity of research examining how public health advocacy is practised, evaluated, and perceived within Australasia.

Ongoing challenges in how public health advocacy is defined, practiced, supported, and evaluated mean that the perspectives of public health advocates offer an important yet underutilized source of evidence. The literature has not routinely captured these perspectives. We surveyed the experiences of Australasian advocates working across diverse settings and fields of public health practice to gain critical insight into current advocacy practices and their perceived effectiveness. Understanding how public health advocacy practitioners define public health advocacy and how they conceptualize the competencies required for effective practice is also essential to further define and strengthen the field. Capturing public health advocates’ perspectives can identify existing competency gaps and inform the design of education, training, and professional development initiatives, thereby strengthening workforce capacity and supporting more effective practice (Sainkhuu *et al*. 2021).

While this study is situated within the Australasian context, the insights generated are likely to be relevant for a global audience, particularly in comparable high-income democratic countries.

Accordingly, this study was guided by the following research questions:

1. How do Australasian public health advocates define and conceptualize public health advocacy?
2. What knowledge, skills, and attributes are perceived as essential for effective public health advocacy practice, and where are key competency gaps within the workforce?
3. What advocacy activities do public health advocates undertake, which strategies are perceived as effective, and what challenges and supports shape advocacy practice?

## Methods

### Study design

A cross-sectional online survey was designed to collect information from public health advocates about how public health advocacy is defined and practiced, which strategies are effective, and the competencies required for practice. The survey was open for a 5-month period between December 2024 and April 2025. Participation in the study was voluntary and anonymous, with written consent obtained electronically prior to survey commencement. A mixed-methods approach was used to provide a more comprehensive understanding of public health advocacy, with quantitative data identifying patterns and qualitative data providing depth and context to participants’ perspectives (Munce *et al*. 2026).

Ethics approval for this study was received from the Medicine, Health and Human Sciences Ethics Committee of Macquarie University (10097/520241009760381), and the study was conducted in accordance with the ethical principles outlined in the Declaration of Helsinki (World Medical Association 2013).

### Participants and recruitment

Participants were self-identified public health advocates based in Australasia and working across the public health workforce. The study focused on Australia, New Zealand, Fiji, and Papua New Guinea, reflecting the membership of the Council of Academic Public Health Institutions Australasia (CAPHIA). The public health workforce was conceptualized broadly to include advocates working across government, academia, non-government, and community organizations. Individuals were eligible to participate if they self-identified as public health advocates, reported any experience undertaking advocacy activities related to the health of communities or populations, and were located in Australasia. This enabled the inclusion of advocates with varying levels of advocacy experience while ensuring participants could meaningfully respond to questions relating to advocacy practice.

Purposive and snowball sampling strategies were used to recruit participants (Moser and Korstjens 2018). Recruitment was undertaken through multiple channels, including peak professional associations, public health organizations, professional networks, and social media.

Invitations to complete the anonymous online survey were distributed via email through the professional networks of the research team, with recipients encouraged to share the survey within their own networks. A follow-up reminder email was sent 4 weeks after the initial invitation. Although the research team’s expertise spans climate and health, epidemiology, monitoring and evaluation, health promotion, workforce development, infectious disease, nutrition, and physical activity, their networks extended across a broad range of public health fields.

To broaden reach, the survey was distributed to public health organizations in Australia, New Zealand, Fiji, and Papua New Guinea. Invitations were addressed either to Chief Executive Officers or sent via general organizational email addresses identified using Google search methods.

In addition, the survey was distributed with a request for advertisement via member newsletters to the Public Health Association of Australia (PHAA), the New Zealand Public Health Association (NZPHA), the Australian Health Promotion Association (AHPA), and the CAPHIA. Available membership information suggested a potential reach of ∼3000 individual members (AHPA and PHAA) and 40 academic institutions (CAPHIA), although membership data were unavailable for NZPHA.

The survey was also publicized on the professional networking platform LinkedIn by the first author.

### Survey design

The research team designed the survey, which comprised 27 questions about participant demographics (gender, age, and location of residence), advocacy experience and practice context, the definition of public health advocacy, advocacy practices and perceived effectiveness, barriers to advocacy and approaches to evaluation, supports, essential public health advocacy knowledge, skills and attributes (competencies), and priorities for future research. In the absence of standardized instruments or agreed public health advocacy competency frameworks, advocacy-related survey items were developed specifically for this study. Question development was informed by the published literature, the Public Health Advocacy Institute of Western Australia’s Toolkit for Advocacy (Stoneham *et al*. 2019), the Australasian public health competency framework (Council of Academic Public Health Institutions Australasia 2016), and consultation with public health advocacy experts, alongside a pragmatic approach to ensure the survey addressed the study aims (Shannon-Baker 2016).

The survey included single choice, multiple choice, and open-ended questions (Supplementary File A), with open-ended items focusing on definition, supports, essential advocacy competencies, competency gaps, and research priorities. With the exception of two advocacy questions (Questions 8 and 12), participants could provide additional detail beyond pre-defined response options. The survey could be completed on either a desktop computer or a mobile device. At the end of the survey, participants were invited to provide their email address if they wished to be contacted regarding participation in a future focus group or interview.

To establish content validity, the survey was piloted with a convenience sample of five public health advocacy experts from Australia and internationally who were not eligible to participate in the survey. Feedback from the pilot informed revisions to the survey.

The survey was administered via LimeSurvey, an online survey management platform.

### Data analysis

Participants were included in the analysis if they completed at least one survey question beyond the demographic items. Quantitative survey data were analysed using descriptive statistics in STATA (version 19.5). Categorical survey data were summarized using frequencies and percentages to describe participant characteristics and advocacy practices (Supplementary File B).

Responses to open-ended survey questions were analysed using a mixed quantitative-qualitative approach informed by applied qualitative methods (Liamputtong 2019). Analysis was guided by *a priori* research questions. The first author undertook an initial reading of all responses to generate preliminary codes, which were refined iteratively through a second reading and discussion with the research team. Coded responses were then grouped into categories, with results presented descriptively as frequencies and percentages to support interpretation. Interpretive description served as the overarching analytic framework, guiding the qualitative analysis in accordance with principles of applied qualitative research and prioritizing practical relevance to health promotion practice (Thorne 2016). Following the initial coding phase, consultation with a researcher experienced in personality psychology informed the categorization of interpersonal skills and attributes to enhance analytical rigour.

QSR NVivo 14 for Windows (QSR, Melbourne, Australia) was used to manage and code qualitative data. Illustrative participant quotations are provided in Supplementary File C.

The qualitative component of the study was reported in accordance with the 21-item Standard Reporting for Qualitative Research checklist (O’Brien *et al*. 2014), included in Supplementary File D. The overall mixed methods study was reported in accordance with the Good Reporting of A Mixed Methods Study checklist (O'Cathain *et al*. 2008), included in Supplementary File E.

## Results

One hundred and thirty-two individuals accessed the survey, with 101 included in the analysis. Demographic details of respondents are summarized in Table 1. Respondents were predominantly female (69%) and based in Australia (90%), with the remaining 10% residing in New Zealand. Among the Australian participants, half (51%) were located in New South Wales.

**Table 1:** Demographic characteristics of survey respondents (*n* = 101).

Characteristic	*n*	(%)
**Gender**		
Female	69	68.3
Male	27	26.7
Non-binary	3	3.0
Prefer not to respond	1	1.0
Missing	1	1.0
**Age**		
18–24	1	1.0
25–34	15	15.0
35–44	25	24.8
45–54	26	25.8
55–64	20	19.8
65 or older	13	12.9
**Location**		
Australia	90	89.1
New South Wales	51	50.4
Queensland	12	11.9
Victoria	9	8.9
Western Australia	8	7.9
South Australia	4	4.0
Australian Capital Territory	4	4.0
Northern Territory	2	2.0
New Zealand	10	9.9
Missing	1	1.0

### Conceptualizations of public health advocacy

The findings in this section are generated from participants’ responses to open-ended survey questions. Illustrative quotes are provided in Supplementary File C, and category frequencies are summarized in Supplementary File B.

#### Definition of public health advocacy

Despite heterogeneity in how participants defined public health advocacy, there was substantial agreement around several core elements, with some including more than one of these elements. Over half of the definitions (57%) included public health advocacy as outcome-focused, and nearly half (49%) indicated improving population health is a defining feature. Around one-third (29%) identified improving policy and practice as central to advocacy efforts, and one-fifth (20%) emphasized that advocacy is focused on addressing health issues, risks, and inequities. Fewer participants described advocacy as focused on increasing awareness of issues (16%), addressing community needs (10%), enhancing equity (9%), securing commitment and resources (9%), and increasing community empowerment and engagement (5%).

Approximately one-third of advocates (35%) indicated public health advocacy involves intentional and strategic actions, with one-fifth (21%) describing it as inherently change-seeking and fewer as evidence-informed (15%). Smaller proportions described advocacy as giving a voice to others (11%), collective efforts (9%), or encompassing a broad range of activities (8%), while few framed advocacy within a human-rights perspective (4%).

About one-third of participants (34%) identified influencing as a key advocacy method commonly targeted at decision makers and those in power, including government (23%) and, to a lesser extent, communities and stakeholders (8%). Around one-fifth of participants (19%) identified communication, particularly informing and educating, as a key method.

*‘Public health advocacy uses strategic collective actions that aim to effectively engage and influence decision makers to take evidence-based actions that deliver better population health outcomes’. P45*

*‘Taking strategic action aimed at influencing social, organisational, and policy changes to improve public health outcomes, including engaging with policymakers, media, and the public to promote evidence-based solutions for population health challenges. Essentially, it means to drive systemic changes for healthier communities’. P39*

#### Competencies for public health advocacy practitioners

##### Essential knowledge for public health advocacy

The most frequently reported essential knowledge for advocacy practice was contextual understanding of the relevant public health issue, identified by over half of participants (63%), which included subject matter expertise (46%) and knowledge of community needs (17%). Smaller proportions of participants identified knowledge of other relevant stakeholders as important (13%).

*‘A good knowledge of the subject area and an understanding of the reasons behind the need for change’. P52*

Policymaking literacy was identified by just over half of participants (53%) as a critical area of essential knowledge. This included an understanding of political processes, policymaking, the structure and function of government, and the political climate surrounding the issue being advocated for. It also included knowledge of who the key decision makers are and an understanding of their interests, motivations, and how to influence them, as well as recognizing the levers of change and identifying windows of opportunity for action.

*‘Knowledge of who the key policy makers are, government departments and how to talk to politicians/decision makers’. P22*

About one-third of participants identified knowledge of public health principles (33%) and advocacy strategy development and methods (30%) as essential, while fewer participants (13%) identified knowledge of industry tactics and opposition and conflict as essential.

*‘Knowledge about the topic of advocacy as well as knowledge about what works to advocate effectively’. P96*

##### Essential skills for public health advocacy

Most participants (87%) identified communication skills as essential for advocacy practice, including written communication (18%) and public speaking (16%). Participants emphasized several aspects of communication as essential, including message framing and tailoring, persuasive communication, storytelling, influencing and negotiation and listening.

*‘Communication skills - media, message framing, storytelling’. P57*

About half of respondents (53%) reported collaborative skills as essential for advocacy practice. This included the ability to engage with others, build and maintain relationships with partners, stakeholders, and decision makers, develop and participate in coalitions, and mobilize communities and network effectively.

Around one-third of participants (32%) identified research skills and media training and social media skills as important (29%).

*‘Ability to work collaboratively, people/networking/communication skills, media skills’. P4*

Although participants were not specifically asked about attributes required for advocacy, interpersonal attributes were identified as important competencies by over half of participants (55%). These included persistence, perseverance, determination, and tenacity (24%), flexibility and agility (11%), passion, drive, and motivation (8%), credibility and integrity (7%), confidence (7%), creativity (5%), and resilience (4%). Interpersonal skills were also identified as essential by around one-third of participants (30%), including being personable, strong listening skills, humour, and empathy.

*‘Perseverance in the face of powerful actors and continued disappointment’. P42*

##### Competency gaps for public health advocacy

Participants were also asked to identify knowledge deficiencies in the current Australasian public health workforce. The most commonly reported were policymaking literacy (19%), effective advocacy strategies (11%), and communicating messages (11%). Commonly identified skill deficiencies were communication (22%), collaborative engagement (18%), and media engagement skills (11%).

### Details of public health advocacy practice

The findings in this section are based on closed-ended survey questions, with the exception of the items on frameworks, tools, and resources for advocacy and priority research areas, which were open-ended. Characteristics of advocacy work and experience are summarized in Table 2.

**Table 2:** Characteristics of advocacy work and experience (*n* = 101).

	*n*	%
**Focus of advocacy work** *		
Healthcare delivery	33	32.7
Climate and health	32	31.7
Equity and inclusion	31	30.7
Environmental health	31	30.7
Alcohol	30	29.7
Tobacco and vaping	29	28.7
Non-communicable diseases	28	27.7
Nutrition	28	27.7
Maternal, child, and youth health	25	24.8
Indigenous health	23	22.8
Public health workforce	23	22.8
Infectious disease	21	20.8
Mental health	20	19.8
Physical activity	20	19.8
Illicit substances	17	16.9
Primary health	16	15.8
Emergency/disaster preparedness	15	14.9
Injury, road, and firearm safety	15	14.9
Housing/homelessness	14	13.9
Domestic and family violence	13	12.9
Gambling	13	12.9
Ageing	10	9.9
Oral health	7	6.9
Occupational health and safety	6	5.9
Other, including sexual and reproductive health, disability, migrant health, sleep health, and cancer screening	9	8.9
**Duration of public health advocacy experience**		
<1 year	4	4.0
1–5 years	20	19.8
6–10 years	52	51.5
>10 years	24	23.8
Missing	1	1.0
**Context of advocacy work**		
Within paid role at an organization	88	87.1
Volunteer for an organization	54	53.5
As an individual	46	45.6
Consultant for an organization	13	12.9
Other, including role within government, student, President of not-for-profit, and pro bono work within role	4	4.0
**Sector of advocacy experience**		
Non-government organization	76	75.2
University or research facility	50	49.5
Independently (not connected to an organization)	30	29.7
Government setting (excluding health settings)	28	27.7
Hospital or healthcare organization	24	23.8
Other, including WHO, volunteer organization, Aboriginal community-controlled health service, specialist medical college, private personal foundation, and community group	6	6.0
**Level of advocacy work**		
Local	86	85.2
National	84	83.2
International	38	37.6
Global	21	20.8

^*^Respondents could select more than one response option.

Respondents reported engaging in advocacy across a range of public health fields, with 85% (*n* = 86) active in more than one field. The most frequently cited fields, each mentioned by more than 20% of participants, included healthcare delivery, climate and health, equity and inclusion, environmental health, alcohol, tobacco and vaping, non-communicable diseases, nutrition, maternal, child, and youth health, Indigenous health, public health workforce, and infectious disease.

Most participants (76%) had 6 to 10 years of advocacy experience or longer. Respondents reported that their advocacy activities were undertaken primarily in a professional capacity as part of paid employment (88%), with half also reporting experience as a volunteer (53%) or as an individual not connected to any specific organization (46%).

Respondents reported advocacy experience across sectors, with the most commonly identified being non-government organizations (NGOs) (75%), university or research settings (50%), independent advocacy not tied to a specific sector (30%), and hospital or healthcare agencies (28%).

Most participants engaged in advocacy at a local (86%) and national (84%) level, while a smaller proportion reported their work included cross-country advocacy activities (international; 38%) or a global (21%) focus.

#### Advocacy strategies implemented

The types of advocacy activities most commonly implemented by participants (Fig. 1) involved writing submissions or commenting on policy briefs (80%), media advocacy (71%), developing coalitions and partnerships (67%), community education (66%), letter writing (66%), policy proposals (62%), and eAdvocacy (59%), which involved the use of digital tools and online platforms to support advocacy efforts. The least commonly reported strategies, though still noted by approximately one-third of participants, were generating debate (34%) and use of champions to support advocacy efforts (36%). Additional strategies (10.9%) identified through open-ended responses and not included in the response options included meeting with politicians and decision makers and stakeholder engagement.

**Figure 1:**
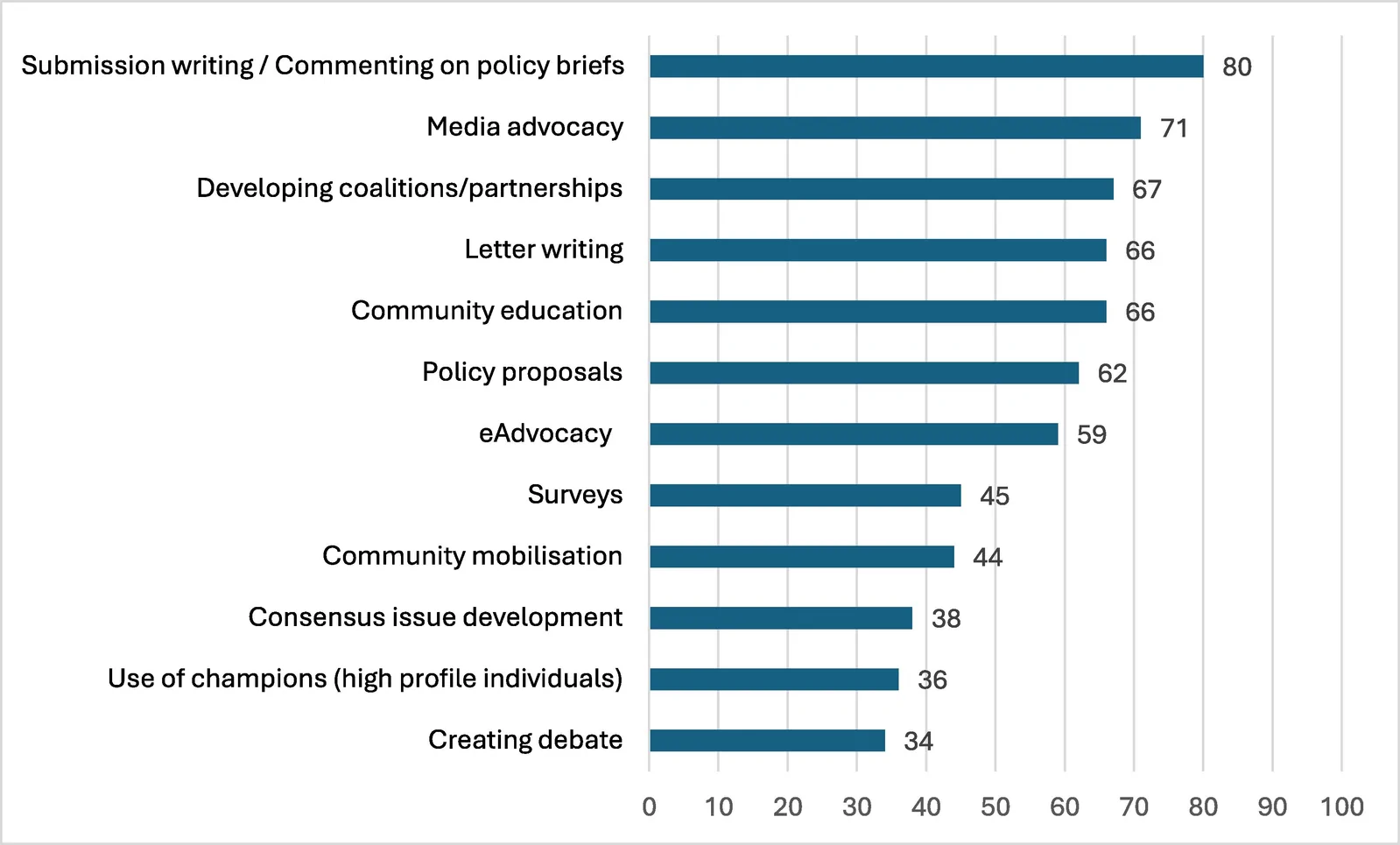
Types of advocacy activity commonly implemented (%).

#### Advocacy targets and motivations

Participants most commonly sought to influence government agencies or officials (91%), community members (76%), and politicians (71%), with half seeking to influence people within their own organization (56%) or their peers (50%), while fewer targeted business owners (21%). Additional targets reported by some participants (11%) were other organizations and media.

Motivations for advocacy were most commonly a passion for the cause (82%) or a sense of professional responsibility (82%), followed by perceived community need (73%). Half of the participants cited alignment with their organizations’ mission (50%) as a motivating factor.

#### Advocacy strategies perceived as effective

Participants selected from a list of options the advocacy strategies they considered most effective or impactful over the past 5 years (Fig. 2). Strategies most commonly identified as effective were developing coalitions or partnerships (59%), influencing politicians or other decision makers (57%) and media advocacy (46%). Approximately one-third of participants also identified community mobilization (34%), community education (32%), and submission writing or commenting on policy briefs (31%) as effective or impactful approaches. In contrast, letter writing and the use of surveys (10%) and creating debate (17%) were viewed as least effective. Nearly half of participants (47.5%) reported other effective approaches in open-ended responses, including relationship building, collaboration with and prioritization of community needs, use of personal stories and storytelling, activism including rallies and strikes, employing lobbyists, contributing to policy and research, petitions, and the use of combined strategies.

**Figure 2:**
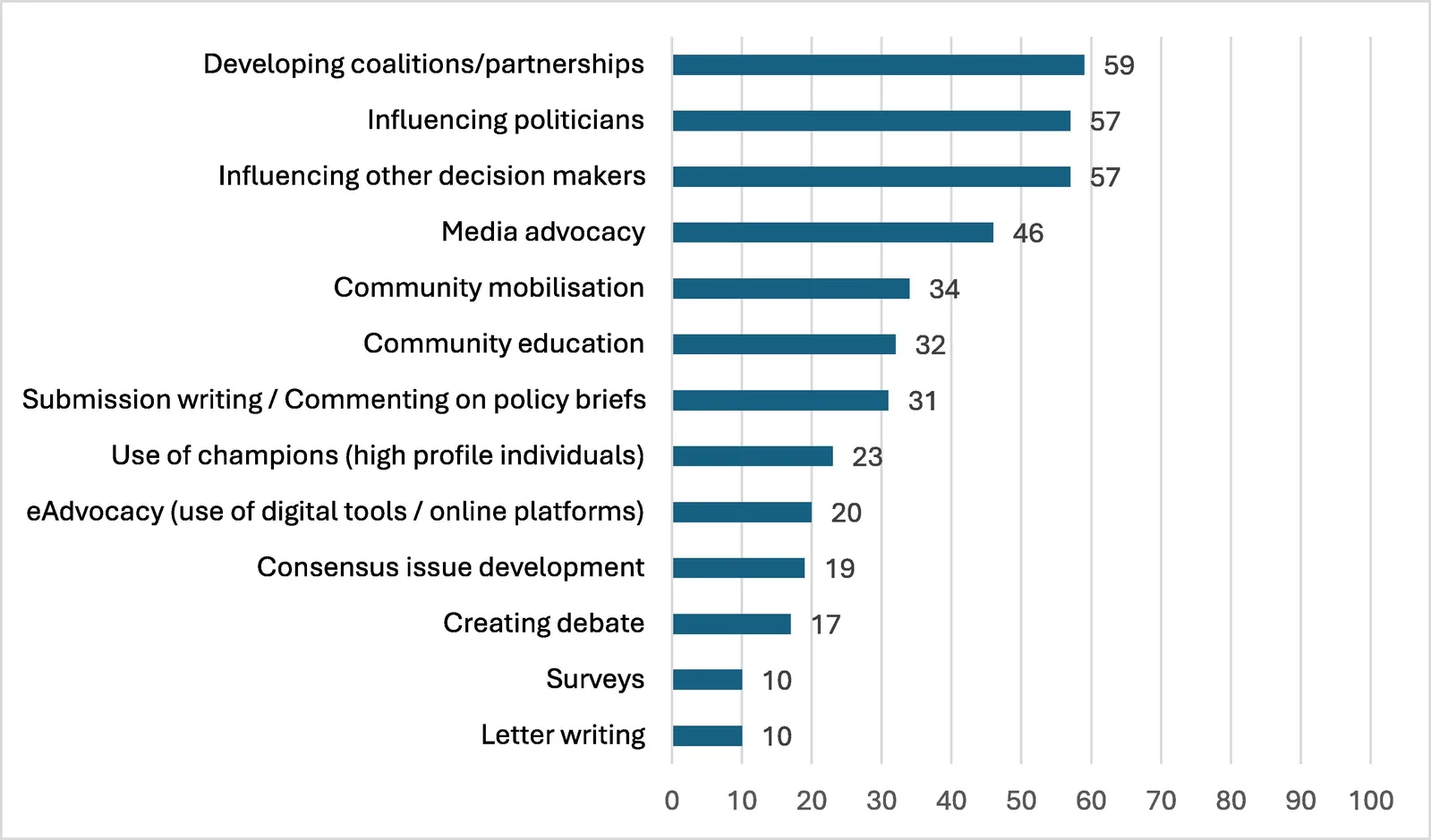
Advocacy strategies perceived as effective (%).

#### Evaluation of advocacy impact

Participants most commonly identified changes to policy as a measure of advocacy impact (66%), followed by media coverage analytics (61%), increased public awareness (54%), and community engagement (49%), while one-fifth (19%) reported that they did not measure their advocacy impact. Other reported measures (9%) included increases in funding, number of activities performed, personal growth in awareness, increase in the use of services, formal project evaluations including impact evaluation, communities of practice, and responses from politicians.

#### Barriers to advocacy practice

Participants identified experiencing several barriers to performing advocacy. The most commonly reported barriers were a lack of funding to support advocacy activities (63%), time constraints (61%), and limited access to decision makers (56%), followed by lack of personnel to support advocacy efforts (47%) and perceived organizational concern about repercussions arising from engaging in advocacy (39%). Around one-third of respondents reported barriers that included limited public support for the issue (33%), low organizational priority of advocacy activities (32%), insufficient advocacy expertise or training and knowledge (32%), personal concerns about repercussions following advocacy activities (28%), and restrictions on advocacy activities (27%). Additional barriers were described by about one-fifth of advocates (18%), including fatigue or burn-out, perceived futility and strong opposition from powerful organizations and industries, internal organizational constraints and bureaucratic processes, challenges within coalitions, misinformation, and a lack of value of lived experience.

Perceptions of repercussions associated with engaging in advocacy varied by organization setting (Table 3). These were most frequently reported by advocates working in government settings (50%), followed by those in hospital or healthcare organizations (46%) and NGOs (40%). In contrast, perceived individual repercussions were most commonly reported by advocates working in universities or research facilities (38%), followed by those in independent organizations (33%) and hospital or healthcare settings (33%).

**Table 3:** Participants’ perceptions of personal or organizational repercussions arising from advocacy activities by organizational setting.

Organizational setting	Perceived personal repercussions	Perceived organizational repercussions
Any setting	28/101 (28.3%)	39/101 (39.4%)
Government setting (excluding health settings)	7/28 (25%)	14/28 (50%)
Hospital/clinic or healthcare organization	8/24 (33.3%)	11/24 (45.8%)
NGO	20/76 (26.3%)	30/76 (39.5%)
University or research facility	19/50 (38%)	16/50 (23%)
Independent organization	10/30 (33%)	10/30 (33.3%)

#### Identified support for advocacy to be effective

Participants were asked about resources or support that would assist in making advocacy activities more effective and identified several key areas. Funding and grants were identified as most needed (74%). Approximately half identified the need for better access to research and data (48%), networking opportunities (47%), an advocacy strategy framework (46%), and advocacy training and workshops (44%). A small number of advocates (8%) identified other supports including time to perform advocacy, recognition of efforts, support from colleagues, and assurances of no repercussions.

#### Frameworks, tools, and resources used for advocacy

In response to an open-ended question about tools, frameworks, and guidelines used to support advocacy, around a quarter of participants (24%) reported not using any, with several participants indicating that while they had a limited awareness of available frameworks, they were interested in learning more about those that exist.

*‘None, just experience but would be good to know if some do exist’. P46*

Research and academic literature were the most frequently reported resources used to support advocacy efforts (18%). Smaller proportions of participants identified specific tools or frameworks, including the Public Health Advocacy Institute of Western Australia’s Toolkit for Advocacy (5%), theory of change (3%), and other planning tools such as advocacy matrices, health communication strategies, and logic models. Learning from others was identified as supportive by some advocates (12%), while others reported learning through experience and trial and error (9%) or via formal training (8%), which was described as limited.

#### Priority research areas

Approximately one-third of advocates (33%) identified evaluation of advocacy initiatives as a priority area for future research. Smaller proportions highlighted the need for research on best practice for community engagement (10%), strategies to address commercial interests and industry influence (9%), and influencing within political contexts (9%).

*‘Measuring the impact of advocacy efforts’. P32*

## Discussion

This study explored how public health advocates from Australia and New Zealand define and conceptualize public health advocacy. It also examined the advocacy practices they engage in and identified the competencies required for effective practice.

### Definition of public health advocacy

Our findings indicate that while participating advocates did not articulate a single shared definition of public health advocacy, their responses identified some common ways in which advocacy was conceptualized. Informed by these recurring elements, the following practitioner-informed working definition of public health advocacy is proposed: *Public health advocacy is an intentional and outcomes-focused practice that uses strategic actions to improve population health. Activities include mobilizing relationships with decision makers, partners, and communities, raising awareness and influencing decision makers to strengthen policy and practice.* This preliminary definition synthesizes the recurring perspectives of participating Australasian advocates and would benefit from further refinement and validation through structured consensus-building methods such as a Delphi study. This practitioner-informed definition aligns with the WHO’s definition of health advocacy as ‘*a combination of individual and societal actions designed to gain political commitment, policy support, social acceptance and systems support for a specific health goal or programme*’ while placing greater emphasis on public health advocacy as an intentional, change-seeking practice and providing greater detail regarding how advocacy is undertaken. Unlike the WHO definition, however, the working definition places less explicit emphasis on gaining political commitment. Participants placed less emphasis on political engagement than anticipated, perhaps reflecting a focus on organizational advocacy, differences in advocacy experience, or less explicit recognition of the political processes underpinning decision-making. The working definition also aligns with the Ottawa Charter’s conceptualization of advocacy as action to create conditions favourable to health (World Health Organization 1986), with participants commonly describing advocacy through the strategic actions they undertake, reinforcing advocacy as an action-orientated component of public health practice. By identifying advocacy activities commonly described by advocates and recognizing advocacy as a relational and collaborative process involving decision makers, communities, and partners, the working definition offers one way of operationalizing public health advocacy and can provide a useful foundation for practice, education, and future research. It also reinforces advocacy as a mechanism for systems change.

These findings contribute to the broader literature on how public health advocacy is conceptualized, by showing that while definitions vary, advocates identify some common conceptualizations alongside considerable heterogeneity. This is consistent with prior research describing fragmented and inconsistently operationalized understandings of public health advocacy across health, legal, political, and social domains, while also emphasizing its close connection to systemic transformation and conceptualizing advocacy in terms of its content, processes, and levels of influence (Monaci *et al*. 2026). However, advocates in our study articulated more consistent conceptualizations of advocacy than those reported in our previous research with Australasian public health educators, who demonstrated more ambiguous and inconsistent understandings (Bhatti *et al*. 2026). This suggests that active engagement in advocacy may support clearer articulation of what advocacy entails in practice, although the operationalization of advocacy is still likely to vary across contexts due to differences in resources and settings. Our findings are also closely aligned with other literature which has highlighted the importance of relationship building, coalitions, and networks in supporting and advancing advocacy practice (Sykes *et al*. 2023, Baker *et al*. 2025, Cullerton *et al*. 2026).

### Required competencies for public health advocacy

In our study, advocates identified key competencies (essential knowledge, skills, and attributes) for public health advocacy practice, including subject matter expertise, an understanding of community needs, policymaking literacy, and familiarity with a broad range of advocacy strategies. Essential skills centred on strong communication and interpersonal capabilities, media, and collaboration and research. Most of these competencies are reflected in the Australasian and WHO European public health competency frameworks, as well as the IUHPE competencies, although they are dispersed across multiple domains and not explicitly framed as advocacy competencies (Council of Academic Public Health Institutions Australasia 2016, World Health Organization 2020, Battel-Kirk *et al*. 2024). Subject matter expertise is not captured in any of the frameworks, interpersonal skills are absent from the Australasian framework, and policymaking literacy is only partially represented in the WHO and health promotion frameworks (Council of Academic Public Health Institutions Australasia 2016, World Health Organization 2020, Battel-Kirk *et al*. 2024). This lack of explicitness highlights a gap in guidance for educators and practitioners seeking to develop advocacy capability.

Several studies have examined the competencies required for effective public health advocacy. A recent international scoping review, closely aligned with our findings, examined how advocacy is conceptualized across public health and healthcare and identified communication and relational abilities as key enablers of effective advocacy, including skills such as storytelling, negotiation, public speaking, and media engagement (Monaci *et al*. 2026). Similarly, research has identified that successful advocates demonstrate strong interpersonal skills, policymaking literacy, determination, resilience, confidence, and organizational permission to act as an advocate (Sykes *et al*. 2023).

Although our study identified policymaking literacy, communication, collaboration, and media skills as essential competencies, advocates in our study also described these areas as persistent gaps within the Australian and New Zealand workforce. Deficits in policymaking knowledge and communication skills identified in our study align with findings from a US study reporting gaps in policy development and communication campaign design (Sainkhuu *et al*. 2021), while gaps in media skills represent a new contribution of our study. This may reflect the rapidly evolving media landscape, where digital and social media have reshaped advocacy practice and require skills that extend beyond those traditionally used. These findings suggest that targeted professional development and curriculum design may help strengthen these capabilities. Gaps in collaborative engagement are also concerning, as uncertainty about how to collaborate effectively may reduce the effectiveness of advocacy efforts.

### Advocacy practices, perceived effectiveness, and evaluation

Our study found advocates use a range of advocacy strategies, with a relatively high proportion reporting the formation of partnerships and coalitions (59%), influencing politicians or other decision makers (57%), and media advocacy (46%) as effective strategies. These Australasian findings are substantially higher than those reported in an international study of 153 advocates across 36 countries in which far fewer advocates identified comparable strategies as effective (Cullerton *et al*. 2026). For example, only 21% reported building relationships with decision makers and influencers as effective, 20% identified coalition-building as effective, and just 8% reported the use of media and social media as effective. One possible explanation for the difference in perceived effectiveness of using media is that the Australasian advocacy context may offer a more conducive media environment for public health messaging than some of the other countries represented in the international study. Interestingly, while media advocacy was identified as effective by almost half of advocates in our study, advocates also highlighted this as an area requiring further strengthening, a finding consistent with previously identified gaps in media-related skills within the Australian public health workforce (McCausland *et al*. 2025).

The most used advocacy strategies in our study were submissions to government and commenting on policy briefs, although only one-third of advocates viewed these as effective. We did not examine the reasons for this limited perceived effectiveness. Few studies have evaluated the effectiveness of submissions as an advocacy approach. Consistent with our findings, a recent Australian study reported only limited short-term policy influence (Ghatalia *et al*. 2023), whereas an earlier study identified submissions as a valuable advocacy strategy (Moore *et al*. 2013). Similarly, while many advocates reported using letter writing (66%), few perceived it as effective (10%). This may reflect its appeal as a simple, low-resource strategy (Hancher-Rauch *et al*. 2019) or as an initial step in the advocacy process compared with more intensive approaches. The term letter writing may also have been interpreted broadly, ranging from public-facing communication, such as opinion editorials and open letters, to direct correspondence with decision makers. Public-facing communication can play an important role in raising awareness, presenting public health perspectives, and contributing to shifts in public discourse and attitudes. These findings raise questions about why advocates invest time in strategies they perceive as relatively ineffective, particularly when strategies perceived to be more effective, such as relationship building with decision makers, were less commonly undertaken. These findings should also be interpreted with the understanding that individual advocacy activities are typically implemented as components of broader advocacy strategies rather than as standalone activities. Notably, influencing decision makers and politicians—despite being perceived as effective—was inadvertently omitted as a response option in our study, limiting assessment of the extent to which this strategy was used.

In our study, when asked how the impact of advocacy activities was assessed, 19% of advocates reported that no measurement was undertaken and 33% identified evaluation of advocacy strategies as a priority research area. Our survey focused specifically on impact measurement rather than the full range of advocacy evaluation approaches, which may also include process, formative, or implementation-focused evaluation. Although impact measurement was reported more frequently than anticipated, this finding should be interpreted cautiously given the use of a limited set of predefined impact indicators. By contrast, existing literature suggests that systematic evaluation of advocacy remains uncommon (Monaci *et al*. 2026). A recent scoping review of advocacy in health and care contexts found that 96% of studies did not report formal evaluation methods and identified a lack of validated and widely adopted evaluation frameworks, limiting the consistency and comparability of evaluation approaches across settings (Monaci *et al*. 2026). Evaluating the effectiveness of advocacy is inherently challenging as advocacy often involves multiple, simultaneous strategies that interact in complex ways, making it difficult to isolate the effects of individual actions (Devlin-Foltz *et al*. 2012, Arensman and Van Wessel 2018). The dynamic and iterative nature of policymaking further complicates efforts to attribute outcomes to specific advocacy activities (Cullerton *et al*. 2018). Advocates may also have limited time and resources to undertake formal evaluations (van Oort *et al*. 2023). Together, these challenges may contribute to the limited use of formal evaluation methods.

### Barriers to advocacy practice

More than half the participating advocates identified limited organizational resourcing and limited access to decision makers as barriers to advocacy. While resource constraints are widely recognized (Wilson *et al*. 2023, Gines *et al*. 2025, Cullerton *et al*. 2026, Marynissen *et al*. 2026), limited access to decision makers represents a novel finding, suggesting that strengthening relational networks may help improve access to decision makers. However, it remains unclear whether this reflects actual barriers, repeated unsuccessful engagement attempts at engagement, or limited understanding of policymaking processes. Future research is needed to clarify these dynamics to inform targeted capacity building efforts.

Consistent with these structural and cultural constraints, one-third of participating advocates reported concerns about organizational or personal repercussions from advocacy activities, aligning with recent UK research identifying fear of consequences as a barrier to practice (Marynissen *et al*. 2026). In our study, organizational concerns were more common in government and clinical settings, suggesting heightened perceived risk. Personal concerns were most frequently reported in university settings, suggesting that stronger institutional support may facilitate academic advocacy. These findings align with evidence that advocates in sectors influenced by strong commercial interests (e.g. tobacco control, alcohol, and ultra-processed foods) often face intimidation or pressure to discourage advocacy (Evans-Reeves *et al*. 2024). Collaborative advocacy initiatives may help mitigate these risks by distributing responsibility, while challenging assumptions that advocacy is inherently confrontational may encourage more practitioners to engage. Strengthening cross-sector collaboration, improving access to decision makers, enhancing evidence use, and ensuring sustainable resourcing are critical to support effective advocacy (Wilson *et al*. 2023).

Limited advocacy expertise was identified as a barrier, with one-third of participating advocates feeling unprepared to engage in advocacy, potentially constraining workforce capacity to influence policy and practice. This is concerning given that participants self-identified as advocates. However, these findings are consistent with previous research identifying advocacy capability gaps and limited professional development opportunities (O'Connell *et al*. 2016, Cohen and Marshall 2017, Sainkhuu *et al*. 2021, Sykes *et al*. 2023, Stoneham *et al*. 2024, Cullerton *et al*. 2026, Marynissen *et al*. 2026).

Approximately one-third of participating advocates reported that advocacy was considered a low organizational priority, aligning with evidence that advocacy is often deprioritized due to perceptions that it is overly political or uncomfortable (Sykes *et al*. 2023, Cullerton *et al*. 2026).

### Strengths and limitations

A key strength of this study was the inclusion of Australasian public health advocates from a wide range of practice fields, enhancing the applicability of the findings to the broader public health community. However, this diversity may reduce the extent to which the findings are applicable to specific fields of public health practice. Other limitations should also be acknowledged. Despite attempts to recruit advocates from Fiji and Papua New Guinea, most participants were based in Australia, predominantly New South Wales, with only a small number from New Zealand. Consequently, the study does not capture the broader regional context, and the sample did not allow for meaningful cross-country comparison. This highlights the need for further country-specific research of advocacy practices to address this gap. Although efforts were made to engage a diverse range of practitioners with advocacy experience, participation was voluntary, and recruitment relied on purposive and snowball sampling, which may have limited participation by advocates outside of established professional networks whose perspectives may have further enriched the diversity of experiences captured. Consequently, the findings should be interpreted as reflecting the perspectives of the participating advocates rather than the broader population of public health advocates. As no standardized instrument was available, a purpose-designed survey was developed for this study. While this enabled the survey to address the study aims, some relevant aspects of advocacy practice may have been omitted, such as political engagement, and the use of a study-specific instrument should be considered when interpreting the findings. Although the mixed-methods design provided depth of understanding, the qualitative component was limited to open-ended survey responses, offering less depth than interviews or focus groups may have provided. The study also explored participants’ perceptions of the effectiveness of various advocacy strategies but did not assess their direct influence on outcomes, indicating the need for research that evaluates the impact of these approaches, an area participants identified as a priority. In addition, competencies identified in this study were compared with selected Australasian and international public health and health promotion competency frameworks. A comprehensive comparison with all existing competency frameworks was beyond the scope of this study and warrants further investigation.

## Conclusion

This study indicates that while public health advocates in Australia and New Zealand articulated diverse definitions of public health advocacy, their descriptions reflected common conceptual elements. Building on these insights, a working definition of public health advocacy is proposed to support clearer conceptualization, more consistent operationalization, and improved comparability across studies. This definition requires further refinement and validation to support broader applicability.

The findings also highlight gaps in workforce capability and organizational support that may constrain effective advocacy. Future research is needed to understand decision makers’ views on what constitutes effective advocacy, which may help practitioners tailor strategies for greater influence. Further evaluation of advocacy strategies is also needed, including the development of frameworks specifically designed to assess public health advocacy activities. The limited quantitative evidence of whether and how advocacy is evaluated in practice highlights a gap in the literature; addressing this through further empirical investigation can strengthen the evidence base for public health advocacy.

### Implications for practice

The findings from this study offer several practical directions for strengthening advocacy capacity, with potential relevance beyond Australasia, particularly in high-income countries with comparable democratic governance and political contexts.

Tertiary public health education could more explicitly embed advocacy competencies, including policymaking literacy, communication, media engagement, and collaborative practice, to better prepare graduates for advocacy roles. Professional development opportunities targeting these areas can also strengthen advocacy capability among the existing workforce.

Organizations can play an important enabling role by creating supportive environments for advocacy, including dedicated time, funding, and resources, as well as reducing perceived risks associated with advocacy and fostering cultures that value advocacy as a legitimate and necessary component of public health work. There is also a need to further articulate and promote the value of advocacy as a core function of public health practice to ensure it is consistently prioritized across sectors.

For advocates, strengthening capabilities in the identified competencies and building relational networks within public health, government, and civil society can improve access to decision makers and enhance the effectiveness of advocacy initiatives. Investing in workforce development and organizational support will better position the public health field to influence the systems, power structures and commercial interests that shape health, advance equitable policy, and improve population health outcomes through effective advocacy.

## Supplementary Material

daag150_Supplementary_Data

## Data Availability

The data underlying this article will be shared on reasonable request to the corresponding author.
