# Supplementary material for: What is public health advocacy? Australasian practitioner perspectives on defining the field and identifying effective practice and required competencies
Source: Health Promot Int. 2026 Oct 21;41(5):daag150. doi: 10.1093/heapro/daag150 (PMC13684274; doi:10.1093/heapro/daag150)
Supplement: Supplementary file 1 [file daag150_supplementary_data.zip › Supplementary File A_Survey Questions.docx]

**Supplementary File A. Public Health Advocacy in Practice Survey**

Survey Invitation

You are invited to participate in this brief survey collecting information from people in Australasia with experience in performing advocacy activities related to the health of communities or populations.

You are encouraged to share this invitation and survey link with others who have advocacy experience.

Who should complete this survey:

Anyone with experience in advocacy activities related to the health of communities or populations from Australasia. This may include but is not limited to advocacy in areas such as climate and environmental health, gambling, or gun control. Insights from people with all levels of experience are valued.

Who should NOT complete this survey:

- Those whose advocacy experience is limited to self-advocacy or advocacy on behalf of an individual or family member.
- Those whose advocacy activities do not relate to population health, (e.g., advocating for a patient).

There are 30 questions in this survey.

**Demographics**

1. **Which gender do you identify as?** Please choose only one of the following:

- Female
- Male
- Non-binary
- Prefer not to say
- Other (please specify):

2. **Please specify your age.** Please choose only one of the following:

- 18-24
- 25-34
- 35-44
- 45-54
- 55-64
- 65 or older

3. **In which location do you live?**  Please choose only one of the following:

- Australia
- New Zealand
- Fiji
- Papua New Guinea
- Other (please specify):

4. **In which state or territory do you live?** Please choose only one of the following:

- NSW
- Victoria
- Queensland
- South Australia
- Tasmania
- Western Australia
- Australian Capital Territory
- Northern Territory

**Public Health Advocacy Questions**

Please answer the following questions from your personal perspective, not on behalf of any organisation you may work for/with. The questions are focused on advocacy activities related to community or population health.

5. **In which context have you had experience in performing advocacy?** Select all that apply.

- Within my paid role at an organisation
- As a volunteer for an organisation
- On behalf of an organisation where I was hired as a consultant
- As an individual who had an interest in the field I was advocating for
- Other (please specify):

6. **Which type of organisation(s) have you worked with or on behalf of when engaging in advocacy activities?** Select all that apply**.**

- Government setting (excluding government health settings)
- Hospital/clinic or healthcare organisation
- Non-government organisation (NGO)
- University or research facility
- Independently (not connected to an organisation)
- Other (please specify):

7. **In which field of public health have you performed advocacy?** Select all that apply.

- Ageing
- Alcohol
- Child and youth health
- Climate and health
- Domestic and family violence
- Emergency / Disaster preparedness
- Environmental health
- Equity and inclusion
- Gambling
- Gun control
- Healthcare delivery
- Housing / Homelessness
- Illicit substances
- Infectious disease
- Indigenous health
- Injury
- Maternal health
- Mental health
- Non-communicable diseases
- Nutrition
- Occupational health and safety
- Oral health
- Physical health
- Primary health
- Public health workforce
- Tobacco
- Vaping
- Other (please specify):

8. **How much experience do you have working in public health advocacy?**  Please choose only one of the following:

- Less than 1 year
- 1-5 years
- 6-10 years
- 10 plus years

9. **What motivates you to engage in public health advocacy?** Select all that apply.

- Personal passion for the cause
- Professional responsibility
- Community needs
- Organisational mission
- Other (please specify):

10. **How would you define public health advocacy?** Please describe
11. **Describe what characterises successful advocacy?**
12. **What level of advocacy have you engaged in?** Select all that apply.

- Local
- National
- International
- Global
- Unsure

13. **Who have you sought to influence with your advocacy activities?** Select all that apply.

- Business owners
- Community members
- Government agencies or officials, e.g. Department Officers
- People within your organisation
- Peers
- Politicians
- Other (please specify):

14. **What type(s) of advocacy activities have you personally performed?** Select all that apply.

- Community education
- Community mobilisation
- Consensus issue development
- Creating debate
- Developing coalitions/partnerships
- E-Advocacy (e.g. use of social media, such as X, Facebook, TikTok, online petitions)
- Letter writing (e.g. Letters to Editor, Open Letter)
- Media Advocacy (e.g. press releases, tv/radio appearances, opinion pieces)
- Policy proposals
- Submission writing / Commenting on policy briefs
- Surveys
- Use of 'Champions' (high profile individuals that advocate for your cause)
- Other (please specify):

15. **In the past 5 years, based on your experience, which types of public health advocacy activities have been the most effective or had the most impact (recognising that specific circumstances may have contributed to their success)?** Select all that apply.

- Community education
- Community mobilisation
- Consensus issue development
- Creating debate
- Developing partnerships/coalitions
- E-Advocacy (e.g. use of social media such as X, Facebook, TikTok, online petitions)
- Influencing politicians
- Influencing other decision makers (e.g. Department Officers)
- Letter Writing e.g. Letters to Editor, Open Letter
- Media advocacy (e.g. press releases, tv/radio appearances, opinion pieces)
- Submission writing / Commenting on policy briefs
- Surveys
- Use of ‘Champions’ (high profile individuals that advocate for your cause)
- Other (please specify):

16. **Are there any other advocacy activities you have found to be effective or impactful not listed above?** Please describe.

17. **What barriers have you faced in your advocacy efforts?** Select all that apply.

- Advocacy activities not regarded as a priority at the organisation you worked for
- Insufficient training or knowledge about advocacy
- Lack of expertise within the organisation
- Limited access to decision-makers
- Lack of funding
- Lack of personnel to support advocacy efforts
- Poor public support for the issue
- Time constraints
- Restrictions on advocacy activities
- Personal worry about repercussions
- Organisational worry about repercussions
- Other (please specify):

18. **How have you measured the impact of your advocacy activities?** Select all that apply.

- Policy changes
- Increased public awareness
- Media coverage
- Community engagement
- Measuring impact was not performed
- Other (please specify):

19. **What resources or support would help you be more effective in your advocacy work?** Select all that apply.

- Training and Workshops
- A comprehensive Advocacy Strategy Framework
- Networking opportunities
- Access to research and data
- Funding and grants
- Other (please specify):

20. **What kind of tools, frameworks or guidelines do you use to support advocacy efforts?** Please describe.

**Knowledge & Skills**

The following three questions relate to your opinion on essential/core public health advocacy competencies.

21. **Describe the essential knowledge required by practitioners to deliver public health advocacy.** Provide as much detail as you can.
22. **From your perspective, what gaps in knowledge related to public health advocacy exist in the current public health workforce in Australasia?** Please describe
23. **Describe or list essential skills required by practitioners to deliver public health advocacy.** Provide as much detail as you can.
24. **From your perspective, what gaps in skills related to public health advocacy exist in the current public health workforce in Australasia?** Please describe.
25. **In the past 5 years, have you been asked to give a university lecture/teaching session on public health advocacy for students?** Please choose only one of the following:

- Yes
- No
- Unsure
- I work at a university and am involved in teaching public health advocacy to students

**Further Research**

It is proposed that the practice of advocacy would be improved by more research into "what works" in successful advocacy. The following questions relate to research related to practice and training in public health advocacy.

26. **Which areas of research into effective advocacy practices should be prioritised to enhance public health advocacy?** Please describe.
27. **What areas of future research on teaching methods and practices for public health advocacy should take priority?** Please describe. *If you don’t have an answer to this question, please leave blank and move to the next question***.**
28. **Can we contact you about being interviewed/part of an online focus group?**  Please choose only one of the following:

- Yes, for a focus group
- Yes, for an interview
- Yes, to both a focus group and interview
- No, to a focus group or interview

29. **Please provide your email address**
30. **Do you consent to data from this study being used to inform subsequent phases of this research/and or in future research projects?** Please choose only one of the following:

- Yes
- No

**Thank you for taking the time to complete this survey.**

If you have any further questions about this survey, please email the research team via alexandra.bhatti@mq.edu.au. If you are not satisfied with the response, you can raise ethical issues or concerns with the human ethics office at Macquarie University via ethics@mq.edu.au.
