# Supplementary material for: What is public health advocacy? Australasian practitioner perspectives on defining the field and identifying effective practice and required competencies
Source: Health Promot Int. 2026 Oct 21;41(5):daag150. doi: 10.1093/heapro/daag150 (PMC13684274; doi:10.1093/heapro/daag150)
Supplement: Supplementary file 1 [file daag150_supplementary_data.zip › Supplementary File B_Category frequencies.docx]

**Supplementary File B: Category frequencies**

| **Category** | % |
| --- | --- |
| **Definition of public health advocacy** | |
| Outcome focused | 57 |
| Targeted at improving population health | 49 |
| Involves purposeful and strategic actions | 35 |
| Influencing is a key method | 34 |
| Involves improving policy, public policy and practice | 29 |
| Activities targeted at decision makers including government | 23 |
| Inherently change-seeking | 21 |
| Addresses health issues risks and inequities | 20 |
| Communication is a key method | 19 |
| Focused on increasing awareness of issues | 16 |
| Evidence informed | 15 |
| Giving a voice to others | 11 |
| Involves collective efforts | 9 |
| Enhances equity | 9 |
| Focused on securing commitment and resources | 9 |
| Targeted at communities and stakeholders | 8 |
| Encompasses a broad range of activities | 8 |
| Focused on increasing community empowerment and engagement | 5 |
| Framed within a human rights lens | 4 |
| **Essential knowledge for public health advocacy** | |
| Subject matter expertise and contextual understanding of the relevant public health issue | 63 |
| Policymaking literacy | 53 |
| Public health principles | 33 |
| Advocacy strategy development and methods | 30 |
| Knowing who the decision makers are and how to influence them | 23 |
| Community context and needs | 20 |
| Working closely with communities | 16 |
| Industry tactics | 13 |
| Relevant stakeholders | 13 |
| Levels of change | 8 |
| Windows of opportunity | 8 |
| Grounding advocacy in lived experience | 4 |
| **Essential skills for public health advocacy** | |
| Communication skills | 87 |
| - written | 18 |
| - public speaking | 16 |
| Collaborative skills | 53 |
| Research skills | 32 |
| Media training and social media skills | 29 |
| Interpersonal skills | 55 |
| - Persistence, perseverance, determination and tenacity | 24 |
| - Flexibility and agility | 11 |
| - Passion, drive and motivation | 8 |
| - Credibility and integrity | 7 |
| - Confidence | 7 |
| - Creativity | 5 |
| - Resilience | 4 |
| Interpersonal skills | 30 |
| **Competency gaps for public health advocacy** | |
| *Knowledge deficiencies* | |
| Government and political processes | 19 |
| Effective advocacy strategies | 11 |
| Communicating messages | 11 |
| Navigating opposition and organisational constraints | 5 |
| *Skill deficiencies* | |
| Communication | 22 |
| Collaborative engagement | 18 |
| Media engagement | 11 |
| **Advocacy targets and motivations** | |
| Government agencies or officials | 91 |
| Community members | 76 |
| Politicians | 71 |
| People within the organisation | 56 |
| Peers | 50 |
| Business owners | 21 |
| Additional targets (other organisations and media) | 11 |
| **Motivations for advocacy** | |
| Passion for the cause | 82 |
| Professional responsibility | 82 |
| Perceived community need | 73 |
| Alignment with organisations mission | 50 |
| **Evaluation of advocacy impact** | |
| Changes to policy | 66 |
| Media coverage analytics | 61 |
| Increased public awareness | 54 |
| Community engagement | 49 |
| Measuring impact was not performed | 19 |
| Other evaluation measures (increases in funding, surveys, number of activities performed, personal growth in awareness, increase in the use of services, formal project evaluations including impact evaluation, communities of practice, responses from politicians) | 9 |
| **Barriers to advocacy practice** | |
| Lack of funding to support advocacy activities | 63 |
| Time constraints | 61 |
| Limited access to decision makers | 56 |
| Lack of personnel | 47 |
| perceived organisational concern about repercussions following advocacy activities | 39 |
| Limited public support for the issue | 33 |
| Low organisational priority | 32 |
| Insufficient advocacy expertise or training and knowledge | 32 |
| Personal concerns about repercussions following advocacy activities | 28 |
| Restrictions on advocacy activities | 27 |
| Additional barriers (fatigue or burn out, perceived futility and strong opposition from powerful organisations and industries, internal organisational constraints and bureaucratic processes, challenges within coalitions, misinformation, lack of value of lived experience) | 18 |
| **Identified support for advocacy to be effective** | |
| Funding and grants | 74 |
| Better access to research data | 48 |
| Networking opportunities | 47 |
| **Frameworks, tools and resources used for advocacy** | |
| Don’t use any | 24 |
| Research and academic literature | 18 |
| Learning from others | 12 |
| Learning through experience and trial and error | 9 |
| Learning via formal training | 8 |
| Public Health Advocacy Institute of Western Australia’s Toolkit for Advocacy | 5 |
| Theory of Change | 3 |
| **Priority research areas** | |
| Evaluation of advocacy strategies | 33 |
| Bes practice for community engagement | 10 |
| Strategies to addresses commercial interests and industry influence | 9 |
| Influencing within political contexts | 9 |
