# Supplementary material for: What is public health advocacy? Australasian practitioner perspectives on defining the field and identifying effective practice and required competencies
Source: Health Promot Int. 2026 Oct 21;41(5):daag150. doi: 10.1093/heapro/daag150 (PMC13684274; doi:10.1093/heapro/daag150)
Supplement: Supplementary file 1 [file daag150_supplementary_data.zip › Supplementary File C_Ilustrative Quotes Table_Revised.docx]

**Supplementary File C. Illustrative quotes table**

| **Domain** | **Theme** | **Illustrative Quote** | **Participant ID** |
| --- | --- | --- | --- |
| Public Health Advocacy Definition | Strategic and intentional actions; Action-orientated; Evidence-based; Outcome focused (population health); Influencing; Decision maker target | "Public health advocacy uses strategic collective actions that aim to effectively engage and influence decision makers to take evidence-based actions that deliver better population health outcomes." | P45 |
|  | Diverse strategies available; Equity-focus; Outcome-focused (population health) | “Various strategies used to ensure equity and improve health outcomes through policy changes.” | P26 |
|  | Diverse strategies available; Outcome focused (improving policy and practice) | “I believe advocacy, at its core, encompasses a wide range of strategies (like communication, negotiation, stakeholder engagement, debate) with the primary aim of improving public health policies to generate better health outcomes within the community.” | P2 |
|  | Evidence-based; Change-seeking; Outcome focused (population health) | “Evidence-based change-making to improve the health of people and planet (they are inextricably linked!)” | P66 |
|  | Evidence-based; Policy, practice and systems | “Actively promoting the uptake and implementation of evidence-based public health policy and practice.” | P5 |
|  | Influencing; Outcome focused (addressing health issue); Decision maker target | “Engaging with key stakeholders with the intention of influencing decisions related to a public health issue.” | P4 |
|  | Influencing; Decision maker target; Policy, practice and systems | “Influencing decision makers (individuals, groups, law makers) regarding behaviour, systems, policy, legislation and/or regulation in order for a positive outcome for my cause.” | P7 |
|  | Influencing; Policy, practice and systems; Outcome focused (raise awareness; population health) | “Efforts to influence policies to invest and promote public health, including raising awareness, capacity building, engaging, mobilizing and empowering communities.” | P12 |
|  | Strategic and intentional actions; Action orientated; Outcome focused; Change-seeking | “The deliberate actions and processes designed to gain public awareness or high-level support to drive systemic change and improve population health outcomes.” | P13 |
|  | Equity-focus | “Work to ensure that there is equitable access to health; human rights are upheld in health system; providing voice to those unable to be present and making sure they are given the opportunity to be present in their own health care.” | P17 |
|  | Policy, practice and systems; Evidence-based; Equity-focus; Evidence-based; Outcome-focused | “Promoting the health and wellbeing of individuals or communities and their right to health and wellbeing - including the right to participate in development of programs, policies and practices that impact them. Advocating for policies, practices and interventions that are evidence-based or informed that can improve health and wellbeing, or advocating for greater access to existing evidence-based initiatives.” | P24 |
|  | Evidence-based; Change-seeking; Policy, practice and systems; Equity-focus, Outcome-focused | “Using data to inform public health investment and policy and programmatic changes to improve health outcomes and health equity.” | P36 |
|  | Strategic and intentional actions; Influencing; Policy, practice and systems; Outcome focused; Evidence-based; Change-seeking; Outcome focused | “Taking strategic action aimed at influencing social, organisational, and policy changes to improve public health outcomes, including engaging with policymakers, media, and the public to promote evidence-based solutions for population health challenges. Essentially, it means to drive systemic changes for healthier communities.” | P39 |
|  | Action-orientated; Influencing; Policy, practice and systems; Outcome-focused | “Involves efforts to influence policies, practices, and behaviours to promote and protect the health and well-being of populations.” | P53 |
|  | Outcome-focused; Policy, practice and systems; Change-seeking | “Working to improve the health and wellbeing of communities through policy, regulation and systems change. It also includes providing a voice for those who are unable to express their views for whatever reason.” | P57 |
|  | Influencing; Decision maker target; Equity focused; Evidence-based | “Influencing decision makers to take up equitable, evidence-informed public health solutions” | P58 |
|  | Outcome-focused; Policy, practice and systems; Diverse strategies available | “About working to improve the health of communities. It focusses on developing and implementing practices and policies to help people to live healthier. It can include a wide range of activities (education, action plans etc) that help people better understand health and healthy living.” | P85 |
|  | Diverse strategies available; Outcome-focused | “All activities that specifically contribute to advocating to various segments of society to advance public health goals” | P88 |
|  | Action-orientated; Influencing | “Undertaking activities aimed at informing and persuading other stakeholders to consider and/or adopt a particular view or approach relevant to public health. Stakeholders include professionals, members of government (politicians and policy makers/public servants), publics.” | P96 |
| Essential knowledge | Contextual understanding of the relevant public health issue | “Knowledge of the topic area of advocacy and the groups you are advocating for.” | P10 |
|  |  | “Thorough knowledge of the topic area.” | P21 |
|  |  | “Subject matter expertise.” | P34 |
|  |  | “A good knowledge of the subject area and an understanding of the reasons behind the need for change.” | P52 |
|  |  | “Very good knowledge of subject matter.” | P71 |
|  |  | “Know your subject - what is the evidence to support what you are asking?” | P86 |
|  |  | “Advocates require a high level of understanding on the issue.” | P91 |
|  |  | “Needs to be anchored in community needs and people with lived-living experience.” | P50 |
|  |  | “Listening to and collating needs from community (verbal, written etc.) and putting it forward to management/ teams to initiative a change.” | P83 |
|  | Policymaking literacy | “Knowledge of the public health issue at large and of policy making/governance.” | P22 |
|  |  | “You have to have a strong knowledge of political topics, and how the Australian parliament works in practice.” | P41 |
|  |  | “Understanding of the political process in Australia. Knowledge of local political environment, such as which groups and individuals have an interest in the issue and how to identify common interests.” | P52 |
|  | Advocacy approaches | “Knowledge of advocacy program design and implementation.” | P69 |
|  |  | “To understand the different kinds of advocacy and how to use them.” | P93 |
|  |  | “What the best strategies are.” |  |
|  | Advocacy methods | “Knowledge of advocacy program design and implementation.” | P69 |
|  |  | “Knowledge of how different advocacy cycles work.” | P99 |
|  |  | “Knowledge about the topic of advocacy as well as knowledge about what works to advocate effectively” | P96 |
|  | Knowledge of decision makers | “Knowledge of who the key policy makers are, government departments and how to talk to politicians/decision makers.” | P22 |
|  |  | “Which department and person is responsible for the policy decision you are trying to influence.” | P60 |
|  | Core principals of public health | “Understanding core principles of public health.” | P27 |
|  |  | “Social, political and commercial determinants of health.” | P42 |
|  |  | “Health promotion, social determinants of health, determination of Health, epidemiology, health policy, participatory methods, emancipatory education, campaign design, strategic planning and evaluation.” | P11 |
| Essential skills | Communication | “Communication skills and the ability to “read” people is important so you can tailor your messaging according to your audience” | P14 |
|  |  | “Communication skills - media, message framing, storytelling.” | P57 |
|  |  | “Clear communication, both in written/visual and oral/audio formats.” | P15 |
|  |  | “Knowledge and practice in being an effective spokesperson.” | P21 |
|  |  | “Communicating effectively and clearly and at a level that suits the audience.” | P27 |
|  |  | “Persuasive writing.” | P32 |
|  |  | “Influencing. Negotiating. Communication (verbal and written)” | P34 |
|  |  | “Writing for different audiences. Developing persuasive, compelling arguments.” | P39 |
|  |  | “How to craft effective messages for different audiences.” | P42 |
|  | Media | “Media skills (including awareness of new media platforms and modes).” | P67 |
|  |  | “Being able to write persuasively, present on traditional media persuasively, and communicate persuasively on a range of social media is also important.” | P37 |
|  |  | “Learn how to engage with media - respond quickly when asked to comment, be succinct while still being accurate.” | P68 |
|  | Collaboration | “Ability to work collaboratively, people/networking/communication skills, media skills.” | P4 |
|  |  | “How to build collaborations with community with lived experience and keep them safe while also then building collaborations with the service provider / decision maker.” | P47 |
|  |  | “Developing partnerships.” | P62 |
|  |  | “Capacity to work with colleagues in coalitions.” | P67 |
|  | Research | “Accessing, understanding and synthesising the evidence - to present an evidence-based approach.” | P39 |
|  |  | “Understanding data and how to communicate it in a simple way.” | P27 |
|  |  | “Research skills - synthesising research evidence.” | P39 |
|  |  | “Critical analysis skills to understand the evidence base.” | P63 |
| Interpersonal attributes | Persistence, perseverance and determination | “Perseverance in the face of powerful actors and continued disappointment.” | P42 |
|  |  | “Persistence. Patience. Impatience.” | P67 |
|  |  | “Determination, resourcefulness, being able to think outside the box.” | P37 |
|  |  | “Stamina and persistence.” | P40 |
| Competency gaps - knowledge | Policymaking literacy | “There is far too little knowledge about government processes; theories of change; and who makes decisions for what reasons.” | P6 |
|  |  | “Understanding of how political decisions are made.” | P27 |
|  |  | “A deep understanding of the political landscape which underpins advocacy efforts.” | P41 |
|  |  | “Lack of awareness of the ‘political world’ and how to access into it” | P71 |
|  | Effective advocacy strategies | “An understanding of the frameworks and proven strategies for how to do advocacy well is needed” | P14 |
|  | Communicating messages | “Knowing how to get the message across via all types of media” | P22 |
| Competency gaps - skills | Communication skills | “Tailored communication skills.” | P23 |
|  |  | “Skills to communicate clearly and succinctly. Skills to influence others.” | P100 |
|  |  | “Influencing. Negotiating.” | P34 |
|  | Collaboration skills | “The networking skills to be able to create relationships with decision makers.” | P41 |
|  |  | “Lack of coordinating, need to work together against incumbent interests (e.g. industries opposing change)” | P35 |
|  |  | “Collaboration, engagement.” | P51 |
|  | Media skills | “Communication and media training.” | P20 |
|  |  | “How to engage with the media (traditional and social).” | P60 |
|  |  | “Media skills (including awareness of new media platforms and modes).” | P67 |
| Frameworks, tools and resources used for advocacy | Limited use of frameworks | “None, just experience but would be good to know if some do exist.” | P46 |
|  |  | “Honestly, none, but would love to learn of them. Most of my work has been on the job learning and common sense. Would be good to understand what works and why, for whom and in what setting.” | P14 |
|  | Research and literature | “Research and literature” | P54 |
|  |  | “Reading up on the latest in the literature” | P49 |
|  |  | “I mostly use research, and my connections with experts in the field” | P2 |
| Priority research areas | Evaluation of advocacy initiatives | “Best practices for stakeholder engagement, addressing health inequities, collaborative teamwork” | P11 |
|  |  | “Measuring the impact of advocacy efforts” | P32 |
|  |  | “Examples of successful advocacy in past and techniques” | P10 |
