# Supplementary material for: What is public health advocacy? Australasian practitioner perspectives on defining the field and identifying effective practice and required competencies
Source: Health Promot Int. 2026 Oct 21;41(5):daag150. doi: 10.1093/heapro/daag150 (PMC13684274; doi:10.1093/heapro/daag150)
Supplement: Supplementary file 1 [file daag150_supplementary_data.zip › Supplementary File E_GRAMMS Checklist_1.docx]

**Appendix E. Good Reporting of A Mixed Methods Study (GRAMMS) Checklist**

|  | **Item** | **Reporting item** | **Checklist** |
| --- | --- | --- | --- |
| **1** | Justification for mixed methods | State the justification for utilising mixed methods in this study. | **✓** |
| **2** | Description of design | Describe the type, priority and sequence of the different methods of study employed. | **✓** |
| **3** | Methods detail | Describe the sampling approach for both quantitative and qualitative data collection and description of data analysis method for both methods. | **✓** |
| **4** | Integration | State how the quantitative and qualitative data was integrated. | **✓** |
| **5** | Method limitations | Identify the limitations associated with combining the two methods used for the study (mixed methods). | **✓** |
| **6** | Insights from integration | Provide details of any new knowledge gained through the integration of quantitative and qualitative data. | **✓** |
